# Spontaneous Twin Ectopic Pregnancy Managed Successfully With Methotrexate-Mediated Ultrasound-Guided Fetal Reduction: A Fertility-Preserving Approach

**DOI:** 10.7759/cureus.17077

**Published:** 2021-08-10

**Authors:** Sparsh Madaan, Arpita Jaiswal, Pankaj Banode, Archana Dhok, Deepika Dewani

**Affiliations:** 1 Department of Obstetrics and Gynaecology, Datta Meghe Institute of Medical Sciences, Jawaharlal Nehru Medical College, Wardha, IND; 2 Department of Interventional Radiology, Datta Meghe Institute of Medical Sciences, Jawaharlal Nehru Medical College, Wardha, IND; 3 Department of Biochemistry, Datta Meghe Institute of Medical Sciences, Jawaharlal Nehru Medical College, Wardha, IND

**Keywords:** ectopic pregnancy, twin ectopic pregnancy, methotrexate, fertility, interventional radiology

## Abstract

Ectopic pregnancy has emerged as an alarming problem for obstetricians worldwide. Live twin ectopic conception is rare to occur, and spontaneously conceived twin ectopic pregnancy is even more infrequent. A 32-years-old gravida 3, para 1, live 1, abortion 2, presented with a confirmation of pregnancy on a urinary pregnancy test kit along with pain in the right iliac fossa. Blood investigations revealed raised serum beta-human chorionic gonadotropin hormone. Transvaginal ultrasonography revealed twin ectopic conception in the right fallopian tube with both the embryos showing cardiac activity and no evidence of intrauterine gestational sac. The patient was managed with methotrexate-mediated ultrasound-guided fetal reduction and is doing well on follow-up presently. Hence, our case report highlights the importance of prompt diagnosis and management of twin ectopic pregnancies with the help of newly evolving interventional radiology procedures.

## Introduction

In an ectopic pregnancy, the implantation occurs outside the uterine cavity post-fertilization, either in singleton or multi-gestational ectopic pregnancies. With emerging tuboplasty and in vitro fertilization, the cases of ectopic conception are on the rise. The ectopic pregnancy is most commonly seen in the fallopian tube (approximately 94%), with 3% in ovaries, and the rest (<1%) are related to abdominal or cervical or in the cornua. The incidence of twin ectopic pregnancies is quite rare and is estimated to be one in 125,000 pregnancies and that of twin tubal ligation to be one in 200 pregnancies [1]. Out of the few reported cases of twin ectopic pregnancies, most were managed surgically, however, very few cases were managed conservatively [2]. While singleton ectopic pregnancies are routinely managed with methotrexate recent advances have shown promising results of methotrexate as a treatment modality even in twin ectopic conceptions. If not managed timely ectopic pregnancy can lead to critical complications. Here, we introduce a rare occurrence of live twin ectopic pregnancy in the right fallopian tube that was successfully managed with methotrexate-mediated ultrasound-guided fetal reduction promptly, thereby preventing maternal morbidity and mortality and also preserving the fertility of the patient.

## Case presentation

**Figure 1:**
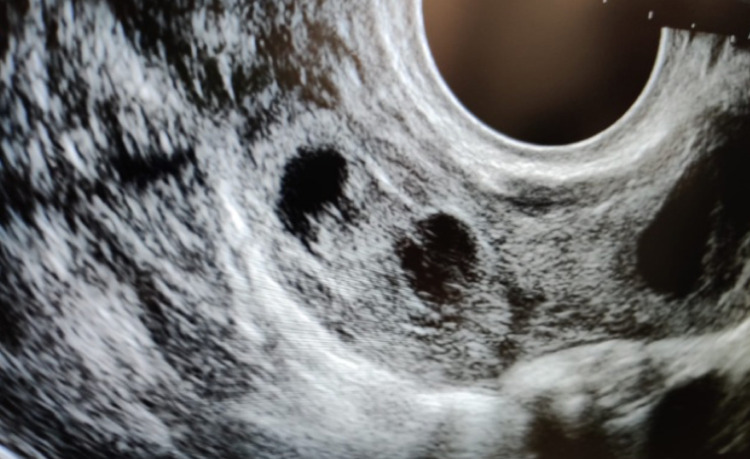
Right adnexal sac with a live twin ectopic gestation of approximately seven weeks and two days. No intrauterine pregnancy.

**Figure 2:**
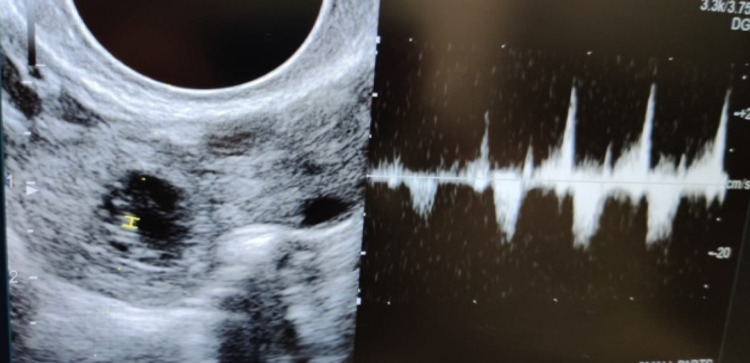
M mode of ultrasonography.

A 32-year-old gravida 4, para 1, live 1, abortion 2 presented with a history of amenorrhoea for one and half months in the outpatient department with urine pregnancy test positive. She presented with complaints of lower abdominal pain for three days and vaginal bleeding. The patient’s gestational age was seven weeks and two days according to her last menstrual period. Prior to her presentation, she had normal regular menstrual cycles. She underwent one lower segment C-section with the outcome of a live female child. Then she had one spontaneous abortion. She had a history of previous ruptured ectopic pregnancy in the left fallopian tube for which she was operated and left-sided salpingectomy was done. She was a non-smoker and had no history of consumption of alcohol. She had no other co-morbidities like diabetes mellitus, hypertension, or tuberculosis. On general examination, the patient was afebrile with vitals stable with a pulse rate of 82 beats per min and blood pressure of 110/70 millimeters of mercury. Her complete blood picture, liver function test, kidney function test, and blood glucose were normal. Serum beta-human chorionic gonadotropin hormone was raised with a value of 10,000 mIU/mL (normal range in the seventh week is 7,650-229,000 mIU/mL). Per abdominal examination revealed uterus of normal size and tenderness was elicited in the right iliac fossa. On per speculum examination, the vagina and cervix were normal with the cervical opening closed and per vaginum examination revealed uterus of multiparous size with cervical motion tenderness and pain elicited in the right fornix. Transvaginal ultrasonography was done, which revealed an anteverted uterus within normal dimensions with no signs of intrauterine pregnancy. Bilateral ovaries were normal and mass of 6 cm with twin ectopic pregnancy with cardiac activity seen in both embryos (Figure 1). Color Doppler and M mode ultrasound (Figure 2) confirmed the presence of cardiac activity in both embryonic poles. The crown-rump length (CRL) of the first twin was 18 mm with the cardiac activity of 122 beats/min, and the CRL of the second twin was 20 mm with a cardiac activity of 128 beats/min, hence, in correspondence to eight weeks and four days of gestational age by Hadlock scale. There was no evidence of free fluid in the pouch of Douglas during the scan. The ultrasound findings suggested live monochorionic diamniotic twin ectopic pregnancy. As the patient had a history of left-sided ruptured ectopic pregnancy and had already undergone left salpingectomy for same, in order to conserve her fertility, the patient was managed conservatively by daycare procedure and underwent ultrasound-guided fetal reduction. In this, with the help of a 23 gauge needle, the yolk sac was punctured percutaneously and 0.5 ml methotrexate was instilled in both sacs. Immediately, there was an absence of cardiac activity in both embryos, and the sacs appeared to be irregular with evidence of hemorrhage within the sacs, thus concluding the procedure.

## Discussion

The fallopian tube carries the ovum, which is produced in the ovaries, to the uterus. An ectopic pregnancy usually occurs in the fallopian tube, and this pregnancy is termed tubal pregnancy. An ectopic pregnancy is a condition that occurs when a fertilized ovum fails to descent in the uterine cavity leading to the growth of fertilized ovum in the fallopian tube. If left unnoticed for a long-time, it may cause bleeding. Such an event might be life-threatening for the patient if it is not treated on time and the patient can present with syncope and signs of shock includes tachycardia, pallor, and collapse. There may be abdominal distension and marked tenderness [3]. Although the incidence of ectopic pregnancy is only 1% of total pregnancies that have been reported, it is considered to be a major hazard for maternal health [4]. In 1986, the first unruptured tubal twin pregnancy was diagnosed by Santos. To date, 106 cases have been described in the literature. However, it is to be noted that this condition is highly underreported because of the diagnosis being mainly surgical or pathological (consider the well-known phenomenon of the vanishing twin and the deterioration of the material after medical therapy). Fewer than 10 cases out of 106 were diagnosed pre-operatively. Very few cases were diagnosed with fetal cardiac activity in a live twin ectopic gestation [5]. Some theories state that delayed ovum transport can cause consequent implantation thereby increasing the chances of monozygotic twin pregnancies [6]. Monochorionic, monoamniotic twin pregnancies will be unilateral. However, if it is dichorionic, diamniotic it may be unilateral but may rarely present as a bilateral ectopic [7]. Contributing factors that can increase the chances of ectopic pregnancy are a history of ectopic pregnancy in the past, pelvic inflammatory disease, artificial reproductive treatment, tuboplasty (tubal recanalization), intrauterine devices, congenital uterine anomaly, and smoking [8]. In this case, the patient presented with intermittent pain in the right iliac fossa, and she was stable with vitals within normal limits. Usually, the treatment for an ectopic pregnancy is based on its clinical presentation, size of G-sac, levels of beta-human chorionic gonadotropin hormone, and complications and may entail conservative, medical, or surgical intervention. The presenting clinical features remain pain in the abdomen and bleeding per vaginum in both singleton and twin ectopic pregnancies making the diagnosis of twin ectopic a difficult one to make on clinical examination and history taking. On blood investigations, there have been no reports suggesting any difference in the levels of human chorionic gonadotropin levels in twin ectopic versus singleton ectopic pregnancies, further complicating the diagnosis of twin ectopic pregnancy to be made even with the help of blood investigations, thus making ultrasonography the only investigation for differentiating twin ectopic pregnancy from singleton ectopic pregnancy. Surgical management is preferred in case of acute ruptured ectopic pregnancy, in hemodynamically unstable patients, in patients with failed medical management, or in those who are contraindicated for medical management. Due to low cost, faster recovery, less time of operation, and short stay at hospital, laparoscopic management is getting preference over other treatment modalities, but the recommended treatment is salpingectomy. However, for women wishing to preserve fertility, salpingostomy can be considered for management. For unilateral tubal twin pregnancies, the surgical approach is usually the treatment of choice according to the literature, similar to singleton ectopic pregnancies. The rate of complications in twin ectopic pregnancy is reported to be more when compared to singleton ectopic pregnancy with chances of rupture in 30 to 50% of the cases [9]. In our case, the patient wanted to preserve fertility and was managed conservatively by ultrasound-guided direct injection of methotrexate into the fetal pole and surrounding gestational sac, and the patient was kept under 24 hours of observation and on antibiotics and was discharged the next day of hospital care. Repeat beta-chorionic gonadotropin hormone was done after 48 hours showing significant decline in level and negative repeat beta-chorionic gonadotropin hormone after seven days. The use of ultrasound-guided methotrexate injection to terminate twin ectopic pregnancy proves that the procedure is as successful as in singleton pregnancy and can be practised to preserve fertility in twin ectopic pregnancy.

## Conclusions

Therefore, we conclude that though twin ectopic pregnancy is rare to find, it should be looked for in ultrasonography, especially in clinical settings suggestive of ectopic pregnancy. Prompt diagnosis of twin ectopic pregnancy and judicious treatment with methotrexate injected with ultrasound-guidance leading to fetal reduction helped to prevent mortality and morbidity along with preservation of fertility in our case.
